# Cerebral Toxoplasmosis in a Renal Transplant Recipient—A Rare Complication

**DOI:** 10.3390/life16030471

**Published:** 2026-03-13

**Authors:** Dubravka Mihaljević, Zvonimir Sitaš, Josip Hanulak, Petar Vranjić, Justina Mihaljević

**Affiliations:** 1 Department of Nephrology, Clinical Hospital Centre Osijek, 31000 Osijek, Croatia; 2 Department of Internal Medicine and History of Medicine, Faculty of Medicine Osijek, Josip Juraj Strossmayer University of Osijek, 31000 Osijek, Croatia; 3 Clinical Institute for Diagnostic and Interventional Radiology, Clinical Hospital Centre Osijek, 31000 Osijek, Croatia

**Keywords:** cerebral toxoplasmosis, immunosuppressive therapy, renal transplant, *Toxoplasma gondii*

## Abstract

Cerebral toxoplasmosis is a rare but potentially fatal opportunistic infection in renal transplant recipients receiving long-term immunosuppressive therapy. It may result from donor-derived transmission or reactivation of latent infection. We report the case of a 70-year-old female who underwent kidney transplantation from a deceased donor in 2004 for end-stage renal disease due to glomerulonephritis. She was maintained on cyclosporine, mycophenolate mofetil, and prednisone. In September 2024, she presented with headache, mood changes, and right-sided hemiparesis. Brain multislice computed tomography revealed a large temporoparietal lesion initially suspected to be glioblastoma. Craniotomy and histopathological analysis demonstrated encysted *Toxoplasma gondii* bradyzoites within gliotic tissue. Polymerase chain reaction testing confirmed the presence of *T. gondii* DNA, while human immunodeficiency virus testing was negative. The patient reported frequent contact with domestic cats. Treatment with pyrimethamine, sulfadiazine, and leucovorin, alongside adjustment of immunosuppressive therapy, led to marked neurological improvement and radiological regression of the lesion. However, nine months later, she succumbed to multidrug-resistant urosepsis. This case highlights the diagnostic challenges of cerebral toxoplasmosis in transplant recipients, as radiological findings are often nonspecific and can mimic neoplastic or lymphoproliferative lesions. Polymerase chain reaction and histopathological analysis remain essential for definitive diagnosis. Awareness of this rare complication is critical for early recognition and prompt initiation of anti-toxoplasma therapy, which can significantly improve outcomes. Although cerebral toxoplasmosis is uncommon after kidney transplantation, it should be considered in immunosuppressed patients presenting with neurological symptoms. Early detection and targeted therapy are key to reducing morbidity and mortality in this population.

## 1. Introduction

Infectious complications remain a leading cause of morbidity and mortality following kidney transplantation and are associated with adverse graft outcomes, including dysfunction, rejection, and graft loss. Both opportunistic pathogens and community-acquired microorganisms contribute substantially to the post-transplant infectious burden. The immunosuppressed state frequently results in atypical or attenuated clinical presentations, complicating timely diagnosis. Kidney transplant recipients are predisposed to severe manifestations of infections, including those caused by organisms typically associated with mild disease. Early and accurate diagnostic assessment is therefore essential to optimize patient and graft outcomes [1].

Up to 70% of recipients experienced at least one infectious episode within the first three years. The timing and spectrum of infections are closely related to the intensity of immunosuppression. In the first month following transplantation, infections are predominantly associated with surgical complications, healthcare-related exposures, and donor-derived pathogens. During this period, multidrug-resistant organisms represent important causes of infection. Reactivation of latent pathogens may also occur, although routine antimicrobial prophylaxis has reduced the incidence of infections caused by Pneumocystis jirovecii, herpesviruses, and hepatitis B virus. Beyond six months post-transplantation, immunosuppressive burden is typically reduced; however, recipients remain susceptible to all types of infections, but especially to cytomegalovirus after discontinuation of prophylaxis. Pre-transplant donor and recipient screening remains essential for infection prevention, while parasitic infections are notably absent from discussion in this otherwise comprehensive review [2].

Organ transplant recipients may acquire parasitic opportunistic infections through three different mechanisms: transmission via the graft, de novo infection, or reactivation of a latent infection as a result of immunosuppression [3]. The prevalence of symptomatic parasitosis infections in renal transplant patients is 2.4%. Most of the infections are caused by Strongyloids stercoralis, followed by Giardia lamblia and, very rarely, *Toxoplasma gondii* and Trypanosoma cruzi [4]. Toxoplasmosis is a globally distributed parasitic disease caused by *Toxoplasma gondii*, an obligate intracellular protozoan capable of infecting all nucleated cells of warm-blooded hosts. Transmission occurs through ingestion of oocysts shed by felids (cats) or consumption of tissue cysts in undercooked meat containing tissue cysts. Serological evidence suggests that approximately one-third of the world’s population has been exposed to the parasite. Infection is usually clinically silent, but there is potentially fatal central nervous system involvement in immunocompromised patients, adverse pregnancy outcomes such as spontaneous abortion and congenital malformations following primary maternal infection, as well as sight-threatening ocular disease in otherwise healthy individuals [5].

Cerebral toxoplasmosis is a life-threatening opportunistic infection most commonly reported in advanced human immunodeficiency virus infection but also occurring in solid organ transplant recipients receiving chronic immunosuppression. In this population, infection may result from donor-derived transmission or reactivation of latent cysts [6]. Whether immunosuppression directly triggers reactivation or permits uncontrolled tachyzoite proliferation remains unclear [7]. Because of the intensive use of immunosuppression in the early post-transplant period, most cases of toxoplasmosis occur within the first three months after transplantation, while late reactivation is rare [8].

Neurological manifestations typically follow a subacute course and may include headache, focal motor deficits such as hemiparesis, cranial nerve involvement, ataxia, seizures, or altered level of consciousness. Serology (anti-Toxoplasma IgG and IgM antibodies) remains the diagnostic gold standard supported by cerebrospinal fluid analysis, histopathology, and neuroimaging. Complete eradication is not currently achievable, as available antimicrobial agents are unable to eliminate *T. gondii* tissue cysts. In patients with neuroimaging findings of multiple cerebral lesions, empiric anti-toxoplasma therapy is recommended regardless of serological status. When there is a high risk of cerebral herniation, surgical biopsy with decompression should be considered. First-line treatment consists of pyrimethamine-based regimens or trimethoprim–sulfamethoxazole, with adjunctive corticosteroid therapy reserved for cases complicated by increased intracranial pressure. The recommended duration of treatment is a minimum of six weeks [9].

Across Europe, pretransplant screening for *T. gondii* is not standardized and depends on individual transplant center policies. Within the Eurotransplant network, routine Toxoplasma serological testing is performed for solid organ donors in only a subset of countries (Croatia included), while others apply a selective testing strategy [10].

## 2. Case Report

We report the case of a 70-year-old female, a retired store worker and mother of two, who lived in a rural area and had lifelong contact with domestic animals, including cats. There was no family history of renal disease. At the age of 16, she underwent a tonsillectomy. At a young age, a renal biopsy was also performed due to proteinuria and hematuria. Histopathological analysis revealed chronic glomerulonephritis. Over the next 20 years, she was regularly followed by a nephrologist for chronic kidney disease. At age 42, she progressed to end-stage renal disease and started chronic hemodialysis. She underwent pre-transplant evaluation and received a kidney from a deceased donor in 2004 at the age of 50. Her comorbidities included arterial hypertension, osteoporosis, and atrial fibrillation. She experienced several hospitalizations for acute deterioration of renal function secondary to enterocolitis, but generally maintained stable graft function on follow-up. Her IgG for anti-Toxo was positive. Information about donor serology remained unknown. Post-transplant, she received standard triple immunosuppressive therapy with cyclosporine, mycophenolate mofetil, and prednisone. Therapy also included prophylaxis with trimethoprim–sulfamethoxazole. She was regularly controlled, and there were no episodes of rejection. Immunosuppression therapy drug levels were monitored, and there were no significant oscillations.

**Figure 1:**
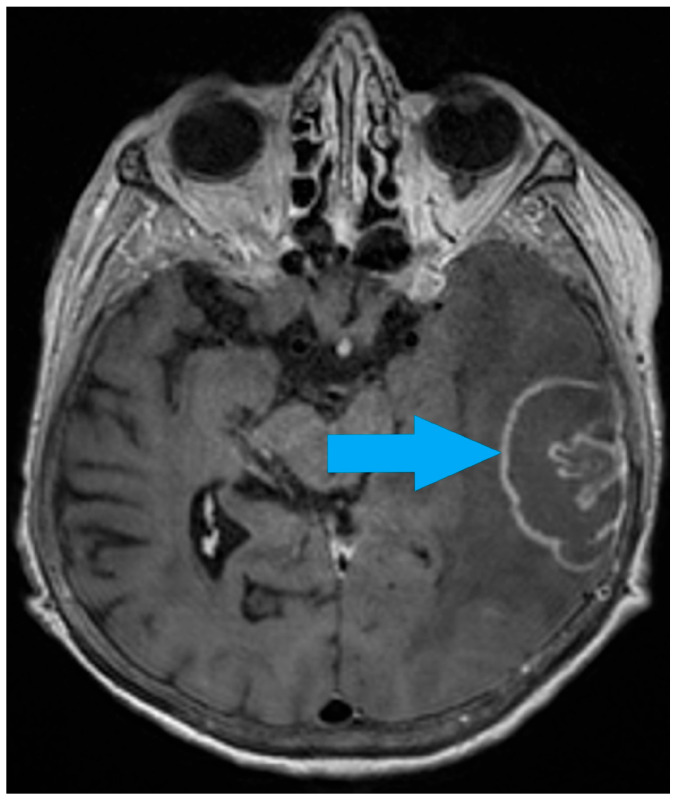
Magnetic resonance imaging (MRI)—a left temporoparietal expansile lesion (55 × 32 × 42 mm) with ring-enhancing post-contrast signal intensity—glioblastoma and features of intratumoral hemorrhage.

In September 2024, she presented with general weakness and was admitted to the Department of Nephrology at the University Hospital Center Osijek, Croatia. The family reported behavioral changes, describing her as withdrawn, apathetic, and excessively demanding. At the time of admission, she was not receiving corticosteroids. The patient complained of a severe headache, a sensation of “pressure in the head,” and mood disturbances. During hospitalization, she developed right-sided hemiparesis. Brain multislice computed tomography (MSCT) revealed an extensive lesion in the left temporoparietal region measuring 68 × 33 × 45 mm, with surrounding edema and midline shift, initially suspected to be glioblastoma. Magnetic resonance imaging (MRI) confirmed a left temporoparietal expansile lesion measuring 55 × 32 × 42 mm with ring-enhancing post-contrast signal intensity, consistent with glioblastoma and features of intratumoral hemorrhage (Figure 1).

A craniotomy was performed with maximal resection of the lesion. Histopathological examination revealed numerous reactive astrocytes (GFAP+), a connective pseudocapsule, and moderate to dense mononuclear infiltration predominantly composed of CD68+ macrophages and CD3+ T lymphocytes. Within the gliotic areas, nodular, granular, basophilic structures consistent with encysted bradyzoites of *Toxoplasma gondii* were identified. Laboratory testing showed stable blood counts without systemic infection, while biochemical results reflected chronic kidney disease. Polymerase chain reaction (PCR) confirmed the presence of *T. gondii* DNA. Serology revealed negative anti-Toxoplasma IgM and positive IgG, while citomegalovirus (CMV) PCR was <100 IU/mL, and HIV testing was negative. Serology was not performed before radiological examinations because the suspicion of cerebral toxoplasmosis was not raised, as it is rare in the late post-transplantation period. Since the radiologists clearly described that it was a glioblastoma, serology was performed only after the pathohistological findings. A diagnosis of cerebral toxoplasmosis was established. The patient was treated with pyrimethamine, sulfadiazine, and leucovorin. Immunosuppressive therapy (mycophenolate mofetil and cyclosporine) was temporarily discontinued, and prednisone was introduced at a dose of 10 mg daily.

**Figure 2:**
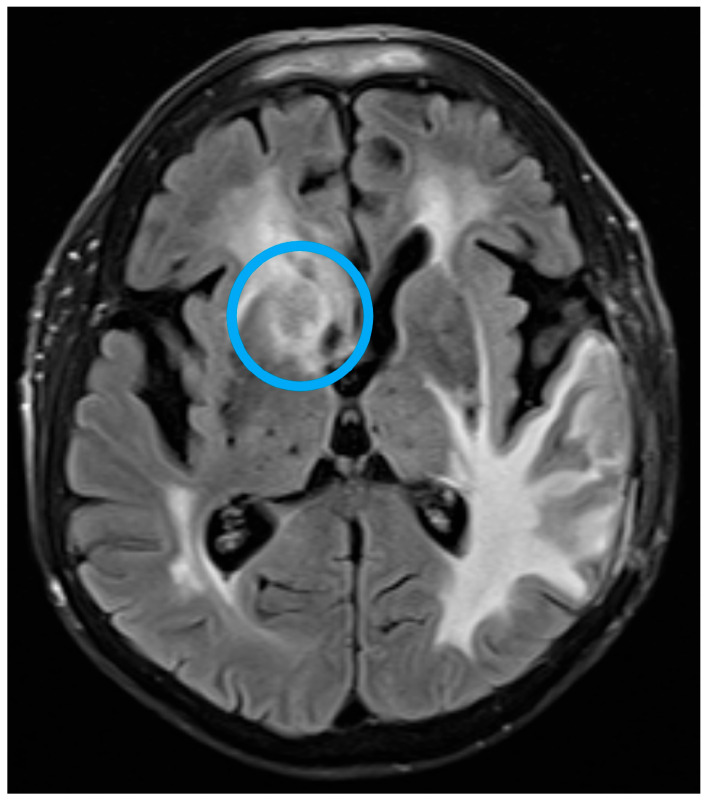
Post-surgical brain MRI—progressive inflammatory lesions consistent with cerebral toxoplasmosis.

**Figure 3:**
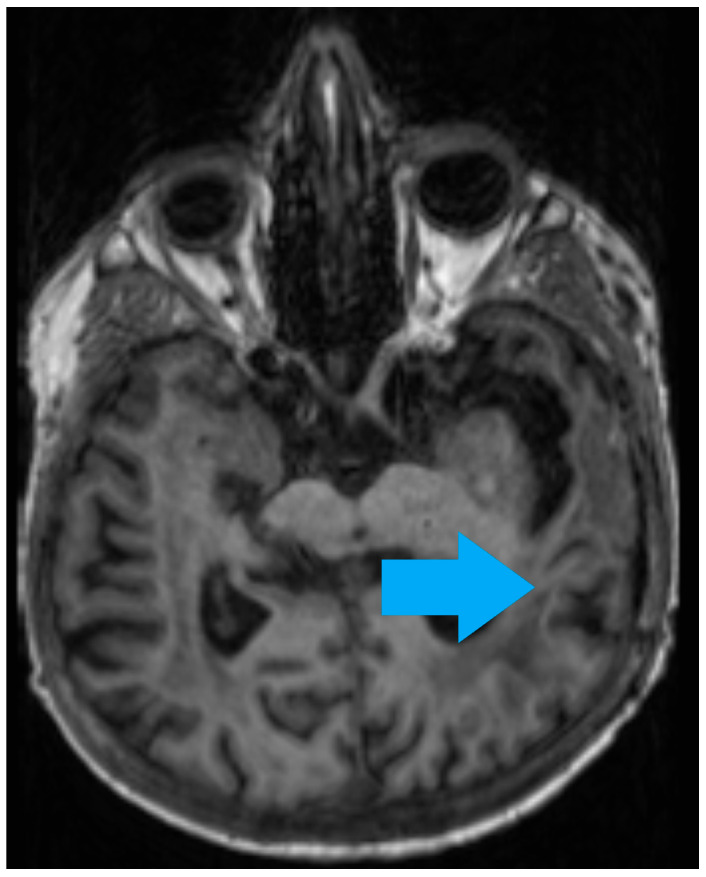
Brain MRI—partial regression of the lesions.

One month after initiation of therapy, brain MRI demonstrated a malacic lesion in the left temporal lobe measuring 28 × 22 mm (post-surgical site) and two newly appearing ring-enhancing foci along the caudal border of the right frontal horn of the lateral ventricle measuring up to 15 × 12 mm, indicating progressive inflammatory lesions consistent with cerebral toxoplasmosis. Clinical improvement was observed within two months, and subsequent neuroimaging showed partial regression of the lesions (Figure 2 and Figure 3).

After ten weeks of antimicrobial therapy, the dose of sulfadiazine was reduced. At fifteen weeks, serology revealed anti-Toxoplasma IgM <3 AU/mL (negative), IgG >650 AU/mL (positive), and CMV DNA PCR 11,391.92 IU/mL (positive). Immunosuppressive therapy was reintroduced in reduced doses. Prednisone was not ceased. The patient was regularly monitored for immunosuppressive therapy doses and was not overly immunosuppressed. The infectious disease team recommended prophylactic trimethoprim–sulfamethoxazole 480 mg once daily for at least six months. Following regression of cerebral lesions, the patient remained in good condition and continued regular nephrological follow-up. In June 2025, she was hospitalized with urosepsis caused by multidrug-resistant Klebsiella pneumoniae and Escherichia coli. Despite intensive antibiotic therapy, the patient passed away due to infection.

## 3. Discussion

Cerebral toxoplasmosis represents a rare but severe opportunistic infection in solid organ transplant recipients. Although it is most commonly described in patients with advanced HIV infection, it has also been recognized in patients who underwent kidney transplantation and received long-term immunosuppression. Most cases occur within the first months after kidney transplantation, when immunosuppressive therapy is at its most intense. However, sporadic cases indicate that reactivation of infection can occur many years after kidney transplantation, suggesting that the risk persists, especially in seropositive individuals [11,12,13].

In the present case, the exceptionally long interval of 20 years between kidney transplantation and disease onset suggests reactivation of latent infection rather than donor-derived transmission. Similar late-onset presentations have rarely been described in the literature. Chandler et al. described disseminated toxoplasmosis occurring 20 years after kidney transplantation, emphasizing the diagnostic challenges of opportunistic infections in long-term transplant recipients [14]. Similarly, Myeong et al. described the development of cerebral toxoplasmosis 21 years after kidney transplantation in a patient with progressive neurological symptoms and multiple intracranial lesions [15]. Other reports describe late-onset symptoms several years after transplantation, further supporting the possibility of delayed reactivation of latent infection in chronically immunosuppressed individuals [16,17]. Despite these similarities, there are several important aspects that distinguish our case from previously reported cases. The neuroradiological findings in this patient strongly suggest a primary brain tumor—glioblastoma. Unlike most other cases, which typically present with multiple intracranial lesions suggestive of infection, our patient presented with a ring-enhanced solitary lesion in the temporoparietal region enhanced with surrounding edema. This tumor-like lesion led to neurosurgical intervention before an infectious etiology was considered.

Magnetic resonance imaging with contrast is the preferred diagnostic modality, but imaging characteristics alone lack sufficient specificity to establish a definitive diagnosis in immunocompromised patients [18].

Second, in our case, the diagnosis was established after neurosurgical intervention with histopathological confirmation and molecular detection of *Toxoplasma gondii* DNA using polymerase chain reaction (PCR). Serological testing has limited diagnostic utility in transplant recipients. While IgM antibodies may indicate recent infection, they are frequently absent in immunosuppressed patients, and IgG positivity merely reflects prior exposure rather than active disease [19]. In our patient, negative IgM and strongly positive IgG supported the hypothesis of reactivation. Molecular diagnostics, such as PCR, may increase diagnostic confidence but demonstrate variable sensitivity and are not universally available [13]. Although previous cases were diagnosed by serology, cerebrospinal fluid analysis, or targeted brain biopsy, in our case, craniotomy and resection of the tumor lesion were performed before diagnosis. Brain biopsy revealed characteristic bradyzoites within inflammatory lesions, providing definitive confirmation.

Standard first-line therapy consists of pyrimethamine, sulfadiazine, and folinic acid, which has demonstrated high efficacy in both HIV-infected and transplant populations [20]. Alternative regimens, including trimethoprim–sulfamethoxazole, may be used in patients with renal dysfunction, hematologic toxicity, or intolerance to first-line agents [21]. Finally, a notable feature of our case is the occurrence of disease in a kidney transplant recipient receiving long-term stable immunosuppressive therapy without recent escalation, which is commonly associated with reactivation of latent infection. However, this case suggests that advanced age, cumulative exposure to immunosuppressive therapy, and patients’ comorbidities may also contribute to infection susceptibility. Following initiation of antiparasitic therapy with leucovorin, sulfadiazine, and pyrimethamine, temporarily with adjustment of immunosuppressive therapy, the patient demonstrated radiological and clinical improvement. A comparison of some previously reported case reports of late toxoplasmosis in kidney transplant recipients is summarized in Table 1. Reduction in immunosuppression is often necessary to enhance immune-mediated parasite control but must be balanced against the risk of graft rejection [12].

Nevertheless, breakthrough infections may occur, particularly after discontinuation of prophylaxis, subtherapeutic dosing, or prolonged immunosuppression. Notably, routine screening for *T. gondii* serostatus in kidney transplant donors and recipients is not universally implemented, potentially contributing to delayed recognition and diagnosis [13]. From a clinical perspective, this case emphasizes several important factors. Cerebral toxoplasmosis should remain part of the differential diagnosis of intracranial masses in kidney transplant recipients, regardless of elapsed time. Furthermore, atypical radiological findings can delay diagnosis, especially if infections are not considered timely. This case underscores the importance of multidisciplinary collaboration among nephrologists, neurologists, neurosurgeons, and infectious disease specialists.

## 4. Conclusions

Cerebral toxoplasmosis remains a rare but potentially life-threatening complication in kidney transplant recipients and may present many years after transplantation. This case illustrates that reactivation of latent *Toxoplasma gondii* infection can occur even under long-term, apparently stable immunosuppressive therapy, posing a significant diagnostic challenge. Clinical presentation and neuroimaging findings are often nonspecific and may closely mimic malignant or inflammatory brain lesions, frequently leading to delayed diagnosis.

Definitive diagnosis requires a high index of suspicion and, in selected cases, histopathological confirmation, particularly when noninvasive diagnostic methods are inconclusive. Prompt initiation of appropriate antiparasitic therapy, together with careful adjustment of immunosuppressive treatment, can result in substantial neurological and radiological improvement. However, the overall prognosis remains guarded due to the high vulnerability of this patient population to subsequent infectious complications.

This case underscores the importance of including cerebral toxoplasmosis in the differential diagnosis of focal neurological symptoms in renal transplant recipients, regardless of the time elapsed since transplantation. Increased awareness, multidisciplinary collaboration, and consideration of preventive strategies may contribute to earlier diagnosis and improved outcomes in this vulnerable population.

## Figures and Tables

**Table 1:** Comparison of reported cases of late cerebral toxoplasmosis in kidney transplant recipients.

Study	Time After Transplantation	Immunosuppressive Therapy	Clinical Presentation	Imaging Findings	Diagnostic Method	Outcome
Present case	20 years	Cyclosporine + mycophenolate mofetil + prednisone	Headache, behavioral changes, right-sided hemiparesis	Solitary temporoparietal ring-enhancing lesion mimicking glioblastoma	Craniotomy with histopathology + PCR	Initial improvement after therapy, death due to multidrug-resistant urosepsis
Chandler et al., 2024 [14]	20 years	Long-term immunosuppressive therapy	Neurological symptoms with intracranial lesion	Enhancing cerebral lesion	Brain biopsy + PCR	Clinical improvement after treatment
Myeong et al., 2022 [15]	21 years	Cyclosporine + mycophenolate mofetil	Cognitive impairment, gait disturbance	Multiple ring-enhancing lesions	Brain biopsy + PCR	Death due to sepsis
Clissold & Bingham, 2010 [16]	6 years	Tacrolimus + steroids + mycophenolate mofetil	Neurological deficits	Multiple cerebral lesions	Imaging + serology	Fatal outcome
Abbas et al., 2020 [17]	15 years	Standard long-term immunosuppression	Fever and neurological symptoms	Multiple cerebral lesions	Serology + PCR	Clinical recovery

## Data Availability

The data presented in this paper are available on request from the corresponding author.
